# Draft genome sequences of four lactic acid bacteria from fermented chicken meat unveil biosynthetic gene clusters for antimicrobial compounds

**DOI:** 10.1128/mra.00038-25

**Published:** 2025-03-10

**Authors:** Mahabub Alam, Tamanna Hassan, Tanvir Ahmad Nizami, Lipi Akter, Tofazzal Md Rakib

**Affiliations:** 1 Faculty of Veterinary Medicine, Chattogram Veterinary and Animal Sciences University, Chattogram, Bangladesh; 2 Faculty of Food Science and Technology, Chattogram Veterinary and Animal Sciences University, Chattogram, Bangladesh; 3 Transboundary Animal Diseases Research Center, Faculty of Veterinary Medicine, Kagoshima University, Kagoshima, Japan; University of Maryland School of Medicine, Baltimore, Maryland, USA

**Keywords:** lactic acid bacteria, fermented chicken meat, *Pediococcus*, *Lactiplantibacillus*

## Abstract

Lactic acid bacteria play a crucial role in fermented food production and serve as important sources of antimicrobial peptides. This study reports four lactic acid bacteria strains isolated from fermented chicken meat, which harbor biosynthetic gene clusters encoding antimicrobial compounds. These strains are classified within the genera *Pediococcus* and *Lactiplantibacillus*.

## ANNOUNCEMENT

Fermented foods have long carried probiotics, prebiotics, and postbiotics. Lactic acid bacteria (LAB) enhance food safety, inhibit spoilage, and support gut health. LAB from the phylum *Firmicutes* include *Lactobacillus*, *Pediococcus*, *Leuconostoc*, *Lactococcus*, and *Enterococcus* (1, 2). With rising antibiotic resistance, bacteriocins are gaining attention as potential alternatives (3). Genome mining and high-throughput screening of biosynthetic gene clusters present a promising pathway for discovering novel bacteriocins, addressing resistance challenges, and advancing sustainable food preservation (4, 5). Three broiler chickens from a live bird market (22.336218 N, 91.830187 E) in Chattogram, Bangladesh were slaughtered under standard procedures. Breast meat (100 g) was stored anaerobically at 4°C for 3 days to propagate probiotic bacteria. Next, 1 g of meat was mixed with 5 mL MRS broth (Oxoid, Hampshire, England) and incubated anaerobically at 37°C for 48 h, promoting LAB growth. Single colonies were isolated on MRS agar, purified, and confirmed by genus-specific 16S rRNA amplification. Genomic DNA was extracted from 1.5 mL of 24 h cultured broth using an AllPrep Bacterial DNA/RNA/Protein Kit (Qiagen, Hilden, Germany), and purity was assessed with a Nanodrop One (Thermo Fisher Scientific, MA, USA). DNA libraries were prepared using the Nextera XT DNA Library Preparation Kit (Illumina, CA, USA). Genome sequencing was conducted on an Illumina NextSeq2000 platform at the Poultry Research and Training Center, Chattogram Veterinary and Animal Sciences University, Bangladesh, generating 2 × 150 bp paired-end reads. Raw data quality, including read quality, GC content, and adapter contamination, was assessed using FastQC v0.12.1 (6). Trim Galore v0.6.5dev (7) was employed to trim low-quality bases and adapter sequences, followed by normalization of read coverage with bbnorm v39.08 (8) to reduce redundancy. *De novo* genome assembly was performed using Unicycler v0.4.8 (9), with subsequent polishing using Pilon v1.24 (10) to enhance accuracy. Genome assembly quality was assessed using QUAST v5.2.0 (11), while Samtools v1.13 (12) was used for alignment file manipulation and indexing. The *de novo* genome assembly conducted through the BV-BRC (13) comprising the abovementioned tools yielded coverage ranging from 65.4 × to 145.6×, with raw reads between 1,452,975 and 1,662,521. The assembled genomes were annotated using the National Center for Biotechnology Information Prokaryotic Genome Annotation Pipeline (PGAP) v6.9 (14) and PATRIC (15) with the in-built RASTtk pipeline to identify the species, with transport proteins identified via TCDB (16), drug targets predicted using DrugBank v6.0 (17), and additional analyses performed for CRISPR arrays and prophages using CRISPRCasFinder (18) and integrative and conjugative elements using ICEFinder (19). All software parameters were set to default, unless specified otherwise. Detailed genomic characteristics are provided in Table 1.

**TABLE 1:** Genomic features of the sequenced four LAB strains isolated from fermented chicken meat in Chattogram, Bangladesh

Strain name Information title	CVASU1	CVASU3	CVASU2	CVASU4
LAB species	*Pediococcus pentosaceus*	*Pediococcus pentosaceus*	*Lactiplantibacillus plantarum*	*Lactiplantibacillus argentoratensis*
Genome submission ID	SUB14898186	SUB14898186	SUB14898218	SUB14898234
Bioproject ID	PRJNA1189463	PRJNA1189463	PRJNA1189463	PRJNA1189463
BioSample accession number	SAMN44971012	SAMN44971014	SAMN44971013	SAMN44971015
SRA accession number	SRR31442436	SRR31442434	SRR31442435	SRR31442433
Genome assembly accession number	GCA_046736495.1	GCA_046736455.1	GCA_046736505.1	GCA_046736475.1
GenBank accession number	JBJPFK000000000	JBJPFJ000000000	JBJPFL000000000	JBJPFM000000000
FastQC Phred score	36	35	37	35
Raw reads	1,452,975	1,925,165	1,600,310	1,662,521
Coverage	113.9×	145.6×	65.4×	70.9×
Contig count	24	26	62	99
Coarse consistency (%)	99.1	99.1	97.9	97.9
Fine consistency (%)	98.2	98.2	96	96.3
Completeness (%)	100	100	100	100
Contamination (%)	1.5	1.5	0	0.6
Genome size (bp)	1,775,093	1,775,155	3,346,512	3,162,126
Contigs N50 (bp)	190,398	174,289	197,047	118,879
Contigs L50	4	4	6	9
Largest contig (bp)	297,937	297,937	471,909	305,211
Guanine–cytosine content (%)	37.153152	37.153095	44.325077	45.074894
Genes (Total), PGAP	1,800	1,799	3,247	3,008
CDS (PGAP; PATRIC)	1,749; 1,783	1,748; 1,783	3,175; 3,353	2,947; 3,108
tRNA	45	45	66	56
rRNA	3	3	3	2
CDS ratio	1.0044544	1.0044193	1.0019387	0.98288304
Hypothetical CDS	350	350	1,507	1,333
Hypothetical CDS ratio	0.29220414	0.29220414	0.50611395	0.49131274
PLFAM CDS	1,700	1,701	0	0
PLFAM CDS ratio	0.95344925	0.95401007	^*a*^-	-
Hypothetical proteins	351	351	1,508	1,334
Proteins with functional assignments	1,432	1,432	1,845	1,774
Proteins with EC number assignments	491	491	687	669
Proteins with GO assignments	407	407	584	561
Proteins with pathway assignments	336	336	488	470
Proteins with subsystem assignments	570	570	716	716
Proteins with PATRIC genus-specific family (PLfam) assignments	1,700	1,701	0	0
Proteins with PATRIC cross-genus family (PGfam) assignments	1,727	1,728	3,233	2,990
Proteins with FIGfam assignments	0	0	0	0
Transporter (TCDB)	2	2	15	8
Drug target (DrugBank)	1	1	1	0
Antibiotic resistance genes (PATRIC)	21	21	25	27
Integrative and conjugative elements (ICEs)	2 (819699..838639, 18,941 bp; 1645942..1683899, 37,958 bp)	3 (819699..838639, 18,941 bp; 1174724..1193031, 18,308 bp; 1662631..1699235, 36,605 bp)	2 (2983152..3034384, 51,233 bp; 3313868..3325625, 11,758 bp)	1 (3096716..3139121, 42,406 bp)
No. of clustered interspaced short repeats (CRISPR) (number of cas clusters) (name of cas genes)	6 (4) (cas9_TypeII, cas1_TypeII, cas2_TypeI-II-III, csn2_TypeIIA)	6 (4) (cas9_TypeII, cas1_ TypeII, cas2_ TypeI-II-III, csn2_ TypeIIA)	2 (1) (cas3_TypeI)	22 (8) (cas6_TypeIE, cas5_TypeIE, cas7_TypeIE, cse2_TypeIE, cse1_TypeIE, cas3_TypeI, cas2_TypeIE, cas1_TypeIE)
Bacteriocins and biosynthetic gene clusters (BGCs)	Pediocin, terpene-precursor, T3PKS	Pediocin, T3PKS	Plantaricin_J, cyclic-lactone-autoinducer, RiPP like, terpene, T3PKS	Terpene-precursor (three loci), T3PKS, terpene

^*a*^ -, not applicable.

The genomes of four LAB isolates were mined for biosynthetic gene clusters (BGCs) associated with antimicrobial production using the software antiSMASH v7.0 (20) and BAGEL4 (21). The genomes were found to contain multiple BGCs encoding ribosomally synthesized and posttranslationally modified peptides (RiPP-like compounds), cyclic lactone autoinducer, type III polyketide synthase (T3PKS), terpene precursors, terpene, and class IIa (Pediocin) and IIb (Plantaricin_J) bacteriocins. The isolates or their purified bacteriocins may be used in the future for food preservation and commercial probiotics to manage gut health and related diseases.

## Data Availability

The accession numbers for the genomes of the four LAB strains are shown in Table 1. All genomes are available under BioProject accession number PRJNA1189463.
